# Exploring the Needs and Expectations of Expectant and New Parents for an mHealth Application to Support the First 1000 Days of Life: Steps toward a Co-Design Approach

**DOI:** 10.3390/ijerph20021227

**Published:** 2023-01-10

**Authors:** Laura Brunelli, Sofia Bussolaro, Raffaella Dobrina, Chiara De Vita, Elena Mazzolini, Giuseppa Verardi, Maura Degrassi, Maria Piazza, Andrea Cassone, Anja Starec, Giuseppe Ricci, Sara Zanchiello, Tamara Stampalija

**Affiliations:** 1 Department of Medical, Surgical and Health Sciences, University of Trieste, 34129 Trieste, Italy; 2 Healthcare Professions Department, Institute for Maternal and Child Health-IRCCS “Burlo Garofolo”, 34137 Trieste, Italy; 3 Area Science Park, 34149 Trieste, Italy; 4 Department of Epidemiology, Istituto Zooprofilattico Sperimentale delle Venezie, 35020 Legnaro, Italy; 5 Obstetrics and Gynecology Clinic, Institute for Maternal and Child Health-IRCCS “Burlo Garofolo”, 34137 Trieste, Italy; 6 Unit of Fetal Medicine and Prenatal Diagnosis, Institute for Maternal and Child Health-IRCCS “Burlo Garofolo”, 34137 Trieste, Italy

**Keywords:** mHealth, pregnancy, app, first 1000 days, co-design, expectant parents

## Abstract

To improve maternal and child health, it is essential to adhere to health-promoting and preventive measures. However, reliable information as well as effective tools are not easy to identify in this field. Our cross-sectional study investigated the needs and expectations of expectant and new mothers and fathers as potential primary users of a hypothetical application supporting the first 1000 days of life. Between May and August 2022, we recruited expectant and new parents by administering an 83-item 5-point Likert scale questionnaire related to the content, functionalities, and technical features of the hypothetical app. We stratified responses using sociodemographic characteristics and then performed ward hierarchical clustering. The 94 women and 69 men involved in our study generally agreed with the proposed content, but expressed low interest in certain app functionalities or features, including those related to the interaction mechanism and interactivity. Women were generally more demanding than men. Our findings, resulting from the engagement of end-users, may be useful for designers and technology providers to implement mHealth solutions that, in addition to conveying reliable information, are tailored to the needs and preferences of end-users in the first 1000 days of life.

## 1. Introduction

The importance of the first 1000 days of life as a unique window of opportunity for improving maternal, newborn, and child health (MNCH) is widely recognized [1,2]. Many international and national institutions are calling for and implementing interventions to support this crucial period [3,4,5,6,7,8], when the foundations of optimum child growth and development across the lifespan are defined, thus impacting future health trajectories and outcomes for individuals. The critical factors that come into play in the first 1000 days of life include stimulation from the earliest possible age, nutrition, and protection from both violence and pollution, among others [3]. To realize the full potential of the mother and child, expectant and new parents should adopt health-promoting measures, such as a healthy diet, adequate physical activity, control of gestational weight gain during pregnancy, abstention from smoking, alcohol, and other substances [8,9], and adherence to preventive health measures such as immunizations and screening [8]. The first 1000 days of life are considered one of the most fruitful time windows for establishing good new health habits and positive behaviors [10]. In view of this, healthcare providers plan and promote educational programs to make new parents aware of anything that might affect their child’s health and wellbeing in the first 1000 days. However, parents themselves have unique information needs during pregnancy, birth, and parenting. The literature, therefore, advocates educational programs tailored to these needs to improve user satisfaction with health services and their effective participation in health-related decision-making [11]. Studies have shown that fathers, in particular, have different challenges and needs related to the perinatal period and that their information and support needs are not fully recognized and met by health services [12,13]. The authors highlight the benefits of interventions to engage fathers in the perinatal period, such as improved communication and decision-making between couples regarding maternal and newborn health, improved care seeking behaviors, increased home health behaviors, and other maternal and newborn health benefits [14]. However, unfortunately, expectant and new fathers are often neglected as the primary target of education and empowerment activities in this particular topic area, and this tendency contributes to their lack of involvement in the baby’s development from the beginning [15,16].

Within this framework, the information available to expectant and new parents may come from a variety of sources whose trustworthiness and reliability are not always easy to determine [17,18]. For these reasons, the health and e-health literacy of individuals largely contributes to the extent to which false information can be acquired or critically analyzed [19,20]. Today, parents and parents-to-be often rely on digital media to obtain information about pregnancy [21], parenting, and childhood [22]. The evidence shows that the quality, accessibility, and affordability of health services have improved following the introduction of telehealth [23]. However, despite the potential key role of telehealth [24,25] and mHealth solutions in providing accurate health information and support in the first 1000 days [26], limited evidence of the effectiveness of these solutions in promoting MNCH has been reported, and many gaps have been identified in available mobile phone applications (apps), in terms of meeting the needs and expectations of both primary (i.e., expectant and new parents) and secondary (i.e., health professionals) users with respect to the content validity [27,28], functionalities, and technical features [28,29]. Nevertheless, the effectiveness of any organizational or technical solution depends heavily on the extent to which it has been shaped by actual users [30,31]. Indeed, it is crucial to adapt and tailor content, functionalities, and technical features to the needs, desires, and expectations of the target audience in order to improve overall user satisfaction, the usability of products and services, and ultimately their effectiveness.

Considering the existing gaps in the literature and the authors’ recent experience in the first steps of the development of a telehealth ecosystem to be tested at the Institute for Maternal and Child Health-IRCCS “Burlo Garofolo” in Trieste (Italy) (henceforth referred to as “IRCCS Burlo Garofolo”), the aim of this study was to investigate the needs and expectations of expectant and new parents as potential primary users of a hypothetical app to support the first 1000 days of life. Specifically, based on all of the above, the present paper tries to seek an answer to the following question: what are the critical information needs of expectant and new parents during the first 1000 days of life?

## 2. Materials and Methods

### 2.1. Data Collection

Between May and August 2022, a convenience sample including both expectant parents in all three trimesters of pregnancy and new parents was recruited to participate in our cross-sectional study. Enrollment took place during scheduled consultations and checkups at the IRCCS Burlo Garofolo carried out either in the course of pregnancy (specifically, before or after ultrasound examination) or in the postnatal period (during hospital stay). Participants with cognitive impairments or without a good understanding of Italian language were excluded.

We administered a questionnaire based on the available scientific literature as well as on the previous experience of the authors who carried out a systematic search for apps related to pregnancy and postnatal care [28,32]. All participants (i.e., both expectant and new mothers and fathers) were informed of the purpose of the study and gave their informed consent to participate in our survey. To ensure confidentiality, the consent form and completed questionnaire were linked to a random code. The questionnaire contained 83 items relating to the six domains of pregnancy care and counselling (26 items), mother and child postnatal care and counselling (13 items), reminders and push notifications (4 items), notes and records (13 items), social support (4 items), and app technical features (23 items). For each item, respondents were asked to indicate the importance of a particular content/functionality/technical feature on a 5-point Likert scale (0—not important at all; 1—of little importance; 2—of average importance; 3—very important; 4—absolutely essential). Participants were also asked multiple-choice questions about the sources/tools they most frequently use to obtain information on pregnancy and postpartum period and the improvements they expected from using an mHealth app specifically designed to support the first 1000 days of life. We also collected data on the sociodemographic characteristics of all participants including age, place of residency (zip code), country of origin, mother tongue, level of education, working condition and hours, and if they were healthcare professionals, their marital status, and family income. Data on the partner, if available (i.e., age, relationship to the coming baby, country of origin, mother tongue, level of education, working condition and hours) and additional data on the current pregnancy and the number of any other children were also collected. Participants completed the questionnaire with paper and pencil in about 15 min and under the general supervision of a researcher who was available if clarification was needed. The Institutional Review Board of the IRCCS Burlo Garofolo formally approved the study (code: IRB-BURLO 08/2021).

### 2.2. Data Analysis

Descriptive statistics on participant sociodemographic characteristics were examined using cross-tabulation and Chi2 test, adjusting with Fisher’s exact statistic when necessary. Correlation was considered statistically significant for *p*-values <0.05. Descriptive analysis was conducted for the ratings reported by expectant and new parents, calculating mean, standard deviation (SD), and frequency for the answers to each item. For the domain analysis, questions with more than seven missing values were excluded (2 of 83 questions were excluded). Observations with more than five missing values among remaining 81 questions were excluded (4 responder exclusions out of 163). After exclusions, 122/159 (76.7%) responders provided full answers to 81 questions. Lastly, 51 overall missing values were replaced using multivariate imputation via chained equations (MICE function with 5 imputed data sets and 10 max interactions). Questionnaire validity and reliability was evaluated with Cronbach’s alpha (0.975 for the 159 questionnaires retrieved for the analysis). Domains values were the average score of domain’s questions score (i.e., the sum of all question scores within one domain divided by the number of questions of the domain). Ward hierarchical clustering was used to classify responders towards their answer patterns. Final number of clusters was selected using the authors’ judgement according to meaningful description of responders’ domain scores. Univariate and multivariate analyses of the outcome variables (mean domain scores and cluster) versus the relevant sociodemographic variables were performed in simple linear regression. Data were analyzed in R 4.1.1 using the R packages mice, ggplot2, dplyr, pvclust, and ltm [33].

## 3. Results

We collected 94 questionnaires from mothers (19 in the first trimester of pregnancy, 21 in the second trimester, 36 in the third trimester, and 16 in the postpartum period; missing = 2) and 69 from fathers (17 in the first trimester of pregnancy, 18 in the second trimester, 16 in the third trimester, and 16 in the postpartum period; missing = 2). The mean age of expectant and new mothers was 33.2 ± 10.4 years, while the fathers’ mean age was 36.1 ± 8.5 years; most participants were Italian (79% women, 91% men). The sociodemographic characteristics of all respondents are shown in Table 1.

When asked about the most commonly used sources of information on pregnancy and the postpartum period, expectant and new parents indicated that they relied most on communities of practice (i.e., informal entities formed by groups of people who share an interest in something that they know how to do and who interact regularly to learn how to do it better [34], such as peer groups, training groups) (*n* = 90; 55%), certified information supported by verified and scientifically validated data sources (e.g., guidelines, services chart) (*n* = 63; 39%), and live communities (i.e., groups of people interested in a particular topic who correspond to each other via a telematic network, such as the Internet or telephone networks, e.g., blogs, forums, online platforms, websites) (*n* = 58; 36%). As shown in Table 2, expectant and new mothers’ and fathers’ opinions were similar in the ranking of these topics, with mothers relying more on live communities and fathers on communities of practice. However, one third of mothers (*n* = 31; 33%) admit that they refer to social media to get the information they need. Moreover, expectant and new mothers cited health professionals (i.e., private gynecologists or midwives), friends, apps, relatives, and friends who already have children, as well as the Internet, as other sources of information used. Expectant and new fathers on their part cited their mother and mother-in-law, scientific articles, books, health professionals, and friends who already have children as additional information tools/sources consulted. Expectant and new parents’ expectations regarding improvements from using an mHealth app for the first 1000 days of life focused primarily on increased preparation and education about pregnancy and the postpartum experience (*n* = 106; 65%), improved communication with health professionals (*n* = 91; 56%), and reduced time spent on education by health professionals (*n* = 80; 49%). These expectations expressed by expectant and new mothers and fathers are detailed in Table 2.

Regarding the desirable information content of an app for the first 1000 days of life, as reported in the Supplementary Materials, expectant and new mothers generally rated as important the proposed content on mother and child postnatal care and counselling, except for information about voluntary abortion which was considered not important or of little importance by 21 women (23%). Expectant and new parents also considered it not so important to include content on violence/abuse during pregnancy (mean score ± SD, 2.6 ± 1.3). Moreover, expectant and new parents’ attention to immunizations recommended for mothers and newborns was low (3.2 ± 1.0). Fathers’ responses, in general, were similar to the mothers’, although they did not place the same importance on hospital baggage (2.5 ± 1.2) as their partners (2.7 ± 1.1). Both groups of parents indicated information about manifest neonatal complications and warning signs as the content with greater importance (3.7 ± 0.7 for mothers and 3.7 ± 0.6 for fathers), accompanied by general information about pregnancy from the fathers’ perspectives (3.6 ± 0.7).

As for the desirable app functionalities and technical features, expectant and new parents considered the app’s ability to set reminders for medical appointments and scheduled medications/immunizations as not very important, thus assigning low scores to this functionality (2.2 ± 1.1 by mothers and 2.1 ± 1.2 by fathers). Regarding the notes and records domain, mothers rated of little importance the app’s ability to record the physiological values of the mother (2.4 ± 1.1) and create a maternal sleep diary (1.6 ± 1.1), functionalities that were also rated on average of little importance by fathers (2.3 ± 1.1 and 1.1 ± 1.2, respectively), as well as the app’s ability to measure maternal weight at baseline and during pregnancy (2.2 ± 0.9) or after birth (2.4 ± 0.9) and keep a sleep diary for the newborn (2.4 ± 0.9). Moreover, the possibility to download data collected through the app, the app’s ability to update users’ account preferences, the use of a simple, informal, and friendly tone by the app, and the presence of certification of the app as a medical device according to Italian law were not highly appreciated by primary users. As for the social support domain, the least appreciated functionality was related to the app’s ability to integrate with social networks (e.g., Facebook and Twitter), which scored 2.3 ± 1.2 for mothers and 1.8 ± 1.1 for fathers. The full list of contents, functionalities, and technical features and their ratings by expectant and new parents are shown in the Supplementary Materials.

**Figure 1:**
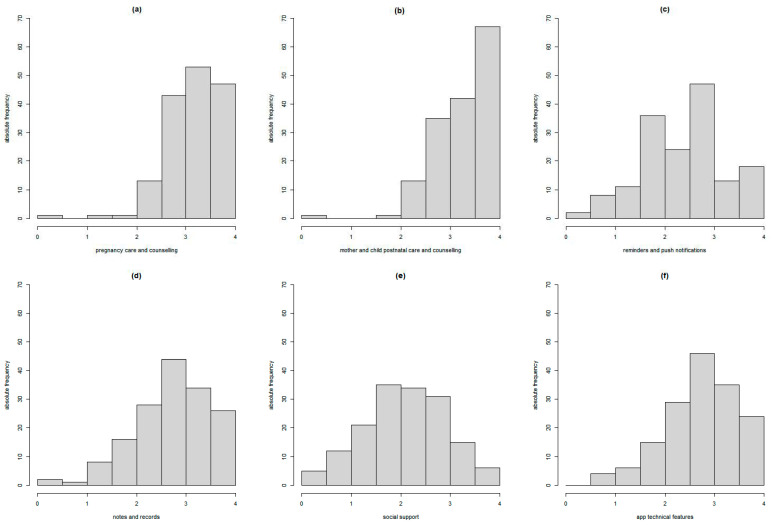
Distribution of the expectant and new parents’ opinions on the content, functionalities, and technical features of an app to support the first 1000 days of life, grouped by the six domains: pregnancy care and counselling (**a**), mother and child postnatal care and counselling (**b**), reminders and push notifications (**c**), notes and records (**d**), social support (**e**), and app technical features (**f**).

The distribution of the mean scores given by primary users to desirable app content/functionalities/technical features, grouped by the six questionnaire domains (i.e., pregnancy care and counselling, mother and child postnatal care and counselling, reminders and push notifications, notes and records, social support, and app technical features), is shown in Figure 1. Overall, higher scores were obtained for the domains of “pregnancy care and counselling”, “mother and child postnatal care and counselling”, and “app technical features”, as shown by the left-skewed distributions of the (a), (b), and (f) plots.

**Figure 2:**
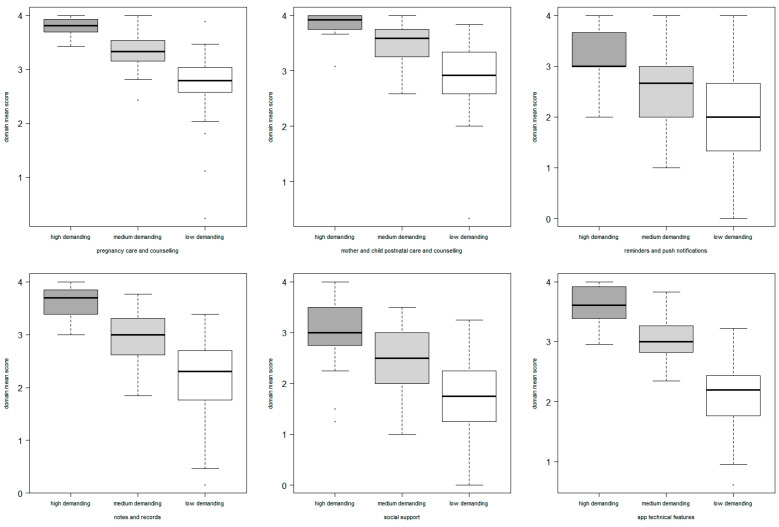
Distribution of responses of expectant and new parents by clusters for each domain presented as boxplots, with the horizontal line in the middle of the box representing the median, the bottom and top the box representing the 25th and 75th percentiles, respectively, and the minimum and maximum of the distribution represented with the upper and lower whiskers.

The surveyed participants were divided into three clusters: cluster#1 comprised 56% of the respondents, cluster#2 comprised 31%, and cluster#3 comprised the remaining 13%. The three clusters identified respondents with high, medium, and low scores for almost all six domains, respectively, as shown in the boxplot of the mean score for the domains in Figure 2.

**Figure 3:**
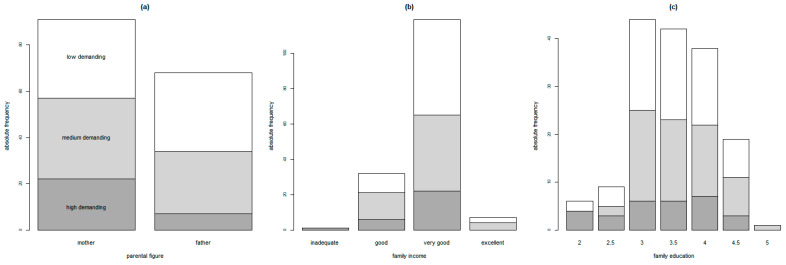
Distribution of primary user clusters, grouped by parental figure (**a**), family income (**b**), and family education (**c**).

After controlling for family income and education, at the multivariate analysis of the composition of the three clusters, we found that women were evenly distributed between clusters, while men were more likely to be found in the low and medium demanding clusters but without reaching statistical significance (*p* = 0.0614) (see Figure 3). No significant differences were found between the three clusters in terms of the average family income or education (*p* > 0.05)

When the mean scores for each domain were analyzed (Table 3), mothers scored higher than fathers in the domains of postnatal care and counselling for mother and child (*p* = 0.002) and social support (*p* = 0.013), whereas respondents with lower income scored higher in the domains of reminders and push notifications (*p* = 0.033) and notes and records (*p* = 0.025). Respondents with lower levels of education scored higher on the variables in the social support domain (*p* = 0.026).

## 4. Discussion

Our study aimed to investigate the needs and expectations of expectant and new parents—also referred to as primary users—towards an app to support the first 1000 days of life. We found a general agreement between both mothers and fathers with the proposed app content, while they expressed skepticism about some app functionalities, especially those related to social support. We did not find a statistically significant association between family income and parents’ perceived needs and expectations in terms of cluster, contrarily to previous studies reporting particularly high information-seeking needs among lower-income pregnant women [35,36], who represent a population at risk of developing pregnancy complications due to potential unequal access to healthcare [37] or inadequate self-education capacities. Indeed, the significant correlation that emerged between family income and the importance accorded to the notes and records, and reminders and push notification domains might suggest a greater need among parents with lower income for external supports to supplement their personal efforts in collecting, monitoring, and keeping track of useful information for their health care. Although no significant difference between clusters emerged in relation to family income, attention must still be paid to the potential difficulties associated with family income, which, in turn, could exacerbate rather than bridge the health divide that makes access to these mHealth solutions difficult due to the cost of the required device (e.g., smartphone, webcam, computer) or due to barriers caused by unstable Internet connections [38,39] in hard-to-reach areas. Although no statistically significant association emerged between family education and parents’ perceived needs and expectations, it is worth pointing out that lower educated parents evaluated the social support domain better than higher educated parents. This result might suggest a greater tendency of lower educated parents to seek support from other people (e.g., people who have gone through similar experiences or medical staff), rather than independently consult certified and validated sources of information, which would require more processing and critical analysis skills. In addition, future and new mothers’ expectations for the proposed mHealth solution were higher than those of future and new fathers. The more demanding nature of mothers may relate to their greater psycho-physical involvement than fathers in pregnancy and the postpartum period.

Empowering patients, citizens, and communities to manage their health has undeniably been one of the main goals since 1986, when the Ottawa Charter for Health Promotion first declared this principle. Yet, as reported by the WHO, there is growing evidence of how individuals’ low health and digital health literacy can impact this empowerment, particularly reducing the capabilities of individuals to actively manage and promote their health and wellbeing [40]. Given these, the observations made in this research regarding the most frequently used information sources leave some doubt. In fact, primary users indicated communities of practice and live communities as usually used, but the reliability and trustworthiness of these sources are not always known, and the assessment of these crucial aspects ends up falling heavily on the shoulders of primary users [28]. However, in light of these considerations, the question of the correct identification of “certified information” by these same users arises [41]. Interestingly, expectant and new mothers, in addition to social media used in one out of three cases, also cited apps as additional sources of information, confirming that the idea of developing an app to support the first 1000 days can, indeed, meet their needs. As Vogels-Broeke and colleagues recently reported [17], more than 50% of women use digital sources during pregnancy, especially nulliparous women. Moreover, the stated expectations of expectant and new parents who participated in our study that using the proposed app will help them increase their preparation and improve their communication with health professionals seem to confirm that this mHealth solution could be a useful tool to bridge the knowledge and language gap between patients and their health professionals [42].

In terms of desirable app content, expectant and new parents paid little attention to vaccinations recommended for mothers and newborns in this study. This situation may reflect some complacency about routine vaccinations that was already present in recent years, as some concerns and misconceptions about vaccine efficacy and safety persist among pregnant women and negatively affect confidence in vaccination [43], but may have worsened after the COVID-19 pandemic [44]. Since the onset of the latter, the delivery of preventive services has, in fact, been adversely affected, with interruptions in routine services and a decline in both access to and demand for routine childhood immunizations all across the country [45]. Regarding pregnant women’s attitude toward vaccination against COVID-19, an mHealth app that provides certified information about the importance and safety of vaccination could increase the confidence of expectant and new parents in vaccination as a means of prevention and health promotion, as it was found that there is a relationship between vaccine acceptance and the way reliable information is provided about the safety and necessity of the vaccine itself [46]. Some interest in prevention was also confirmed by our expectant and new parents focusing their attention on neonatal complications and warning signs that might precede the onset of more serious health disorders in their children. In addition, as stated above, our expectant fathers reported a high level of interest in general information about pregnancy, confirming their need and desire to learn about what is happening in the bump and to visualize the unborn child, as has already been described in the literature [15,47]. In contrast, interest towards content about violence and abuse during pregnancy and voluntary abortion was low. These findings could be indicative of parents’ lack of interest in issues that, although widely current and widespread, do not directly concern their life experience.

In general, the participants’ attention to the content related to pregnancy and postnatal period underscores expectant and new parents’ awareness of numerous issues denoting the complexity and diversity of their needs during the first 1000 days of life. Nonetheless, participants rejected the proposal of an app that provides reminders for routine activities and a sleep diary for mother and child and likely express disagreement with the proposal that the app strictly controls their lives. Being additionally burdened by the “pushiness” of information not expressly sought can indeed further exacerbate the level of stress that expectant and new parents may already experience during the first 1000 days of life [48]. In some contrast to the desktop design [28], expectant and new parents also rejected the use of the proposed mHealth solution in conjunction with their social media, possibly calling for a sharper separation between fun or social support and formal healthcare. Although participants also expressed less interest in app technical features than researchers expected, some doubts remain, as some of these proposed technical elements may be considered minimum standards for any app, such as screen orientation adaptability. However, some skepticism towards the app’s ability to use geolocation may also stem from the user’s concern to provide personally identifiable and sensitive information which can be stored, transmitted, and accessed beyond the user’s control. The constant monitoring of geolocation by an app has indeed been identified as a feature that encourages user’s mistrust [49] to the detriment of potential benefits that this functionality can offer in terms of the identification of and connection with health and social services within a given area.

As discussed by other colleagues, digital solutions should be viewed as complements rather than substitute tools [50]. However, to ensure that these mHealth solutions are effective, it is essential that both primary and secondary users are involved in their development to meet the information needs of today’s generation of expectant and new parents [17,30]. This co-design process, to ensure maximum participation and empowerment of parents and the best outcomes for the child as well, should also take into account that the parental profiles of mothers and fathers may differ not only in terms of the content they need but also in terms of their alignment to motherhood and fatherhood frames [47].

This study has several limitations that should be considered to better interpret our findings. First, we collected data from expectant and new parents attending only the IRCCS Burlo Garofolo, precluding us from exploring the opinions of expectant and new parents in other rural contexts. Moreover, as we used a convenience sample, our findings may indeed be prone to selection bias linked to reasons regarding accessing the Institute on data collection days, which were unknown to the research group. Nevertheless, the Institute considered in the study is the only health facility that provides public antenatal care in the urban and suburban area, so we can assume that the sample is representative of the target population. Second, we used a self-report questionnaire to survey parents’ opinions, so possible bias due to social desirability cannot be excluded as, for example, the low information need expressed by women regarding violence-related content. Nevertheless, we assured participants that their responses would be fully anonymized to control for this risk. Furthermore, we cannot exclude that participants in the survey were more sensitive and aware of the usefulness of reliable health information and, therefore, even more motivated to contribute to this study. In addition, as the questionnaires were administered to participants in potentially tense situations (e.g., before the ultrasound examination or during the hospital stay), the answers given by parents may have been somewhat influenced by their level of anxiety or a certain amount of carelessness. Moreover, we chose to use only a quantitative research method to explore primary users’ needs and expectations regarding a large number of different aspects related to the use of a hypothetical mHealth app to support the first 1000 days of life (i.e., app content, functionalities, and technical features, as well as expectations regarding improvements from using such an app). However, we do not rule out the possibility of extending the findings of the present study in the future by further exploring some of the elements highlighted in this cross-sectional study through a qualitative investigation involving primary users (e.g., by conducting interviews and/or focus groups). Finally, in this study, we only explored the needs and expectations of primary users, i.e., expectant and new parents, without considering the perspective of secondary users, i.e., health professionals. However, additional research could include the opinions of health professionals to develop even more comprehensive and effective mHealth solutions to support the first 1000 days of life.

It is also worth mentioning the strengths of our study. Firstly, the involvement, by design, of primary users in the process of developing an app to support them in the first 1000 days of life gives expectant and new parents the opportunity to actively contribute to shaping an information tool (i.e., an app) to promote their healthcare in this crucial stage of life. Moreover, by involving both expectant and new mothers and fathers, we were able to explore the different points of view and needs of both women and men on the parenting process in support of a gender equality perspective. Moreover, the results of our analysis can easily be translated into operational indications useful for the effective design and development of user-focused mHealth tools, thus revealing relevant practical implications.

## 5. Conclusions

Our findings, resulting from a co-design approach with primary end-users of an app supporting the first 1000 days of life, provide several useful indications for the development of mHealth solutions corresponding to the needs and expectations of new and expectants parents, paying attention not only to mothers, who are classically considered in previous studies, but also to fathers, who are generally neglected in the literature but equally involved in the parenting process. These insights highlight how crucial it is to involve healthcare organizations in defining and sharing reliable and certified information content capable of responding to the pressing education needs of our target audiences in a gender-sensitive manner. Similarly, our results can be useful guidelines for designers and technology providers to match the technical features and functionalities of implemented mHealth tools to mothers’ and fathers’ practical needs during pregnancy and the postnatal period. In this framework, the synergy of the expertise of health professionals and technology developers serving the needs of patients/users can be seen as a generator of tailored mHealth tools and services capable of effectively supporting parents and children during the first 1000 days of life.

## Figures and Tables

**Table 1:** Sociodemographic characteristics of the participants.

Variable		Participant			
		Expectant and New Mother (N = 94)		Expectant and New Father (N = 69)	
		Mother	Partner	Father	Pregnant Partner
**Age (years); Mean ± SD**		33.2 ± 10.4	35.7 ± 5.3	36.1 ± 8.5	32.8 ± 4.5
		***n* (%)**	***n* (%)**	***n* (%)**	***n* (%)**
Place of residence	Trieste—city center	53 (56%)	/	36 (52%)	/
	Trieste—suburbs	29 (31%)	/	23 (33%)	/
	Other	10 (11%)	/	8 (12%)	/
	(missing)	2	/	2	/
Country of origin	Italy	74 (79%)	77 (82%)	63 (91%)	65 (94%)
	Other	20 (21%)	17 (18%)	6 (9%)	0
	(missing)	0	0	0	4
Mother tongue	Italian	75 (80%)	76 (81%)	64 (93%)	66 (96%)
	Other	19 (20%)	18 (19%)	5 (7%)	0
	(missing)	/	/	/	3
Level of education	Primary school	0	1 (1%)	0	0
	Lower secondary school	5 (5%)	9 (10%)	5 (7%)	3 (4%)
	Upper secondary school	35 (37%)	49 (52%)	36 (52%)	29 (42%)
	University	45 (48%)	27 (29%)	22 (32%)	32 (46%)
	Post-university	9 (10%)	8 (9%)	6 (9%)	5 (7%)
Working condition	Manager, Businesswoman/businessman	7 (7%)	6 (6%)	5 (7%)	4 (6%)
	Freelance professional	5 (5%)	12 (13%)	10 (14%)	4 (6%)
	Employed	51 (54%)	44 (47%)	32 (46%)	42 61%)
	Worker	8 (9%)	29 (31%)	22 (32%)	8 (12%)
	Housewife/housemaker	11 (12%)	0	0	4 (6%)
	Unemployed	10 (11%)	1 (1%)	0	7 (10%)
	Other non-specified	2 (2%)	1 (1%)	0	0
	(missing)	/	1	/	/
Working hours (only for working participants)	Full-time	50 (68%)	90 (96%)	69 (100%)	47 (81%)
	Part-time	23 (32%)	2 (2%)	0	11 (19%)
	(missing)	0	1	/	0
Healthcare professional	Yes	19 (20%)	/	4 (6%)	/
Pregnancy	Singleton	89 (95%)	/	67 (97%)	/
	Multiple—twins	3 (3%)	/	1 (1%)	/
	(missing)	2	/	1	/
Type of conception	Planned	26 (28%)	/	35 (51%)	/
	Unplanned	62 (66%)	/	31 (45%)	/
	Assisted reproduction	5 (5%)	/	3 (4%)	/
	(missing)	1	/	0	/
Professional caregiver assisting the current pregnancy	Midwife (public service)	26 (28%)	/	19 (28%)	/
	Private gynecologist	50 (53%)	/	42 (61%)	/
	Other	18 (19%)	/	8 (12%)	/
Other children	None	49 (52%)	/	45 (65%)	/
	One	30 (32%)	/	17 (25%)	/
	Two	11 (12%)	/	7 (10%)	/
	Three or more	4 (4%)	/	0	/
Marital status	Married or with a partner	93 (99%)	/	68 (99%)	/
	(missing)	1	/	1	/
Family income	Upper	3 (3%)	/	5 (7%)	/
	Middle	63 (67%)	/	51 (74%)	/
	Sufficient	23 (24%)	/	12 (17%)	/
	Insufficient	1 (1%)	/	0	/
	(The respondent preferred not to answer)	4 (%)	/	1 (1%)	/
Biological father of the coming baby as current partner (only for mothers)	Yes	92 (98%)	/	/	/

**Table 2:** Summary of responses from participants on the most frequently used information sources and expected improvements resulting from the use of an app to support the first 1000 days of life.

Variable	Expectant and New Mothers (N = 94)	Expectant and New Fathers (N = 69)
Most used information sources *; *n* (%)		
Community of practice (e.g., peer groups, training groups)	48 (51%)	42 (61%)
Live communities (e.g., blogs, forums, online platforms, websites)	38 (40%)	20 (29%)
Certified information (e.g., guidelines, service charts)	36 (38%)	27 (39%)
Social media (e.g., Facebook, Twitter)	31 (33%)	15 (22%)
Digital communication tools (e.g., WhatsApp)	8 (9%)	3 (4%)
Expected improvements from the use of a dedicated mHealth app *; *n* (%)		
Increased preparation of pregnant women/new mothers	61 (65%)	45 (65%)
Improved communication between expectant/new parents and health professionals	51 (54%)	40 (58%)
Reducing the amount of time spent providing information by health professionals	45 (48%)	35 (51%)
Use of a common code	9 (10%)	6 (9%)

* Multiple answers allowed.

**Table 3:** Correlation between main outcomes (cluster and domain mean score) and main parental sociodemographic variables; correlation estimates are calculated in multivariable linear regression model.

	Cluster (High, Medium, Low Demanding)		Domain Mean Score											
			Pregnancy Care and Counselling		Postnatal Care and Counselling for Mother and Child		Reminders and Push Notifications		Notes and Records		Social Support		App Technical Features	
	Corr	*p-*Value	Corr	*p-*Value	Corr	*p-*Value	Corr	*p-*Value	Corr	*p-*Value	Corr	*p-*Value	Corr	*p-*Value
Mother (baseline) vs. father	0.25	0.039	−0.10	0.268	−0.29	0.002	−0.23	0.079	−0.18	0.137	−0.33	0.013	−0.03	0.78
Family income	0.12	0.327	0.03	0.744	0.04	0.703	−0.29	0.033	−0.28	0.025	−0.08	0.576	0	0.975
Family education	0.06	0.528	0.05	0.465	0.12	0.104	−0.10	0.338	−0.10	0.283	−0.23	0.026	−0.05	0.604

## Data Availability

The data presented in this study are available as Supplementary Materials and upon reasonable request to the corresponding author.

## Supplementary Materials

The following supporting information can be downloaded at: https://www.mdpi.com/article/10.3390/ijerph20021227/s1, Figure S1: Contents, functionalities, and technical features and their ratings by expectant and new parents.
