# Supplementary material for: Exploring the Needs and Expectations of Expectant and New Parents for an mHealth Application to Support the First 1000 Days of Life: Steps toward a Co-Design Approach
Source: Int J Environ Res Public Health. 2023 Jan 10;20(2):1227. doi: 10.3390/ijerph20021227 (PMC9858695; doi:10.3390/ijerph20021227)
Supplement: Supplementary file 1 [file ijerph-20-01227-s001.zip › ijerph-2095054-supplementary.pdf]

Domain	Item	Expectant and new parents (N=159)												Expectant and new mothers (N=91)												Expectant and new fathers (N=68)											
		M	SD	0		1		2		3		4		M	SD	0		1		2		3		4		M	SD	0		1		2		3		4	
				n	%	n	%	n	%	n	%	n	%			n	%	n	%	n	%	n	%	n	%			n	%	n	%	n	%	n	%	n	%
Pregnancy care and counselling	General information about pregnancy (e.g., nutrition/weight gain, fitness, oral health, travel health)	3.5	0.8	2	1	0	0	15	9	39	25	103	65	3.4	0.9	2	2	0	0	10	11	26	29	53	58	3.7	0.6	0	0	0	0	5	7	13	19	50	74
	Information about drugs that can be taken during pregnancy	3.4	0.9	3	2	5	3	11	7	50	31	90	57	.3.3	0.9	1	1	4	4	5	5	34	37	47	52	3.4	0.9	2	3	1	1	6	9	16	24	43	63
	Information about vitamin supplement during pregnancy	3.1	0.9	1	1	4	3	36	23	61	38	57	36	3.1	0.9	1	1	3	3	19	21	35	38	33	36	3.1	0.8	0	0	1	1	17	25	26	38	24	35
	A list of free-of-charge and upon payment examinations to be carried out during pregnancy	3.5	0.7	1	1	1	1	17	11	48	30	93	58	3.4	0.8	1	1	1	1	10	11	28	31	52	57	3.5	0.7	0	0	0	0	7	10	20	29	41	60
	Information about infections that can occur during pregnancy (e.g., toxoplasmosis, cytomegalovirus)	3.5	0.8	3	2	0	0	11	7	48	30	97	61	3.4	0.9	3	3	0	0	8	9	27	30	53	58	3.6	0.6	0	0	0	0	3	4	21	31	44	65
	Information about the immunizations that the mother needs to receive during pregnancy	3.2	1.0	5	3	1	1	28	18	52	33	73	46	3.2	1.0	4	4	1	1	11	12	30	33	45	49	3.1	0.9	1	1	0	0	17	25	22	32	28	41
	Information about voluntary pregnancy interruption	2.5	1.3	15	9	21	13	37	23	44	28	42	26	2.5	1.3	10	11	11	12	19	21	28	31	23	25	2.5	1.3	5	7	10	15	18	26	16	24	19	28
	Information about spontaneous miscarriage	3.0	1.0	6	4	7	4	28	18	59	37	59	37	3.0	1.0	3	3	4	4	14	15	36	40	34	37	2.9	1.1	3	4	3	4	14	21	23	34	25	37
	Information about violence/abuse during pregnancy	2.6	1.3	15	9	14	9	39	25	43	27	48	30	2.6	1.3	10	11	7	8	19	21	25	27	30	33	2.5	1.2	5	7	7	10	20	29	18	26	18	26
	Information about possible problems related to pregnancy	3.3	0.9	4	3	3	2	18	11	46	29	88	55	3.3	1.0	4	4	2	2	7	8	26	29	52	57	3.3	0.8	0	0	1	1	11	16	20	29	36	53
	Information on fetal development	3.6	0.7	1	1	1	1	8	5	47	30	102	64	3.5	0.8	1	1	1	1	7	8	23	25	59	65	3.6	0.5	0	0	0	0	1	1	24	35	43	63
	Information about fetal movements	3.3	0.9	3	2	4	3	21	13	53	33	78	49	3.4	0.9	3	3	0	0	6	7	31	34	51	56	3.1	0.9	0	0	4	6	15	22	22	32	27	40
	Information about prevention measures during pregnancy (e.g.,	2.9	1.0	4	3	8	5	39	25	53	33	55	35	2.9	1.1	3	3	6	7	19	21	28	31	35	38	2.9	0.9	1	1	2	3	20	29	25	37	20	29

	home accidents, car accidents)																																				
	Information about labour	3.5	0.8	3	2	1	1	10	6	45	28	100	63	3.6	0.9	3	3	1	1	2	2	20	22	65	71	3.4	0.7	0	0	0	0	8	12	25	37	35	51
	Information about delivery (e.g., epidural anaesthesia, caesarean section)	3.5	0.8	2	1	2	1	12	8	34	21	109	69	3.6	0.8	2	2	2	2	2	2	19	21	66	73	3.5	0.7	0	0	0	0	10	15	15	22	43	63
	Information about cord blood donation	2.9	1.0	4	3	11	7	39	25	55	35	50	31	2.9	1.0	3	3	4	4	21	23	32	35	31	34	2.8	1.0	1	1	7	10	18	26	23	34	19	28
	Information about pre-delivery courses	3.0	0.9	1	1	8	5	32	20	60	38	58	36	3.2	0.9	1	1	3	3	14	15	35	38	38	42	2.9	0.9	0	0	5	7	18	26	25	37	20	29
	Information about in-hospital stay for mother and newborn at the time of delivery	3.0	0.9	2	1	8	5	32	20	57	36	60	38	3.2	0.9	2	2	3	3	12	13	33	36	41	45	2.8	0.9	0	0	5	7	20	29	24	35	19	28
	Information about the woman’s rights during pregnancy (e.g., at work, at school, economical support)	3.3	0.9	1	1	8	5	17	11	46	29	87	55	3.4	0.9	1	1	3	3	7	8	24	26	56	62	3.2	0.9	0	0	5	7	10	15	22	32	31	46
	Information on prenatal risks and life-threatening conditions for both the mother and the foetus during pregnancy	3.4	0.8	2	1	3	2	10	6	58	36	86	54	3.4	0.9	2	2	3	3	2	2	32	35	52	57	3.4	0.7	0	0	0	0	8	12	26	38	34	50
	Information about maternal physiological and metabolic changes occurring during pregnancy	3.1	0.9	1	1	5	3	33	21	66	42	54	34	3.1	0.8	1	1	3	3	13	14	43	47	31	34	3.0	0.9	0	0	2	3	20	29	23	34	23	34
	Information about maternal or child services accessibility and contacts	3.2	0.9	1	1	3	2	28	18	53	33	74	47	3.3	0.8	1	1	2	2	10	11	32	35	46	51	3.1	0.9	0	0	1	1	18	26	21	31	28	41
	Information about available prenatal diagnostic tests	3.3	0.8	2	1	3	2	18	11	61	38	75	47	3.3	0.9	2	2	1	1	11	12	28	31	49	54	3.2	0.8	0	0	2	3	7	10	33	49	26	38
	Information on physical exercises and workouts for women during pregnancy	2.8	0.9	0	0	8	5	55	35	54	34	42	26	2.9	0.9	0	0	6	7	23	25	35	38	27	30	2.7	0.9	0	0	2	3	32	47	19	28	15	22
	Month/trimester-related tips for pregnant women	3.2	0.8	1	1	5	3	20	13	75	47	58	36	3.2	0.8	1	1	3	3	7	8	41	45	39	43	3.0	0.8	0	0	2	3	13	19	34	50	19	28
	A list of essentials for the hospital luggage	2.7	1.1	7	4	19	12	33	21	59	37	41	26	2.9	1.1	4	4	6	7	17	19	36	40	28	31	2.4	1.1	3	4	13	19	16	24	23	34	13	19
other	A list of essentials for the	3.2	0.9	2	1	6	4	24	15	50	31	77	48	3.4	0.8	1	1	2	2	9	10	27	30	52	57	3.0	1.0	1	1	4	6	15	22	23	34	25	37

	first welcome of the mother and baby at home																																				
	Information about maternal physiological and metabolic changes occurring during the postpartum period	3.3	0.8	1	1	2	1	23	14	51	32	82	52	3.4	0.8	1	1	1	1	8	9	30	33	51	56	3.2	0.8	0	0	1	1	15	22	21	31	31	46
	Information about manifest neonatal complications and warning signs	3.7	0.7	1	1	2	1	5	3	28	18	123	77	3.7	0.7	1	1	2	2	2	2	17	19	69	76	3.8	0.5	0	0	0	0	3	4	11	16	54	79
	Information about postpartum mental disorders. such as postpartum depression and baby blues (e.g., symptoms and coping strategies)	3.4	0.8	1	1	2	1	18	11	47	30	91	57	3.5	0.8	1	1	2	2	5	5	28	31	55	60	3.3	0.8	0	0	0	0	13	19	19	28	36	53
	Tips for the postpartum recovery process	3.3	0.7	0	0	1	1	22	13	59	37	78	49	3.5	0.7	0	0	1	1	6	7	28	31	56	62	3.1	0.7	0	0	0	0	15	22	31	46	22	32
	Information on breastfeeding practices	3.4	0.8	1	1	0	0	21	13	54	33	83	52	3.5	0.7	1	1	0	0	6	7	26	29	58	64	3.1	0.8	0	0	0	0	15	22	28	41	25	37
	Information on mother's nutrition after childbirth and during breastfeeding	3.2	0.9	1	1	5	3	26	16	59	37	68	43	3.3	0.8	1	1	1	1	13	14	32	35	44	48	3.0	0.9	0	0	4	6	13	19	27	40	24	35
	Practical tips on how to take care of the newborn (e.g., hygiene, diapers' changing, and burping)	3.3	0.8	2	1	1	1	16	10	64	40	76	48	3.5	0.7	1	1	0	0	4	4	33	36	53	58	3.1	0.8	1	1	1	1	12	18	31	46	23	34
	Information about neonatal screening procedures	3.3	0.7	0	0	0	0	24	15	63	40	72	45	3.5	0.6	0	0	0	0	7	8	34	37	50	55	3.1	0.8	0	0	0	0	17	25	29	43	22	32
	Information about the immunizations that mothers or newborns need during the first 1,000 days	3.4	0.7	0	0	1	1	19	12	55	35	84	53	3.5	0.7	0	0	1	1	7	8	29	32	54	59	3.3	0.7	0	0	0	0	12	18	26	38	30	44
	Information about risk prevention measures regarding the newborn	3.4	0.7	1	1	1	1	14	9	63	40	80	50	3.5	0.7	1	1	0	0	5	5	34	37	51	56	3.3	0.7	0	0	1	1	9	13	29	43	29	43
	Information on methods for postpartum family planning and birth spacing	2.7	1.0	2	1	18	11	45	28	51	32	43	27	2.9	1.0	2	2	7	8	20	22	32	35	30	33	2.5	1.0	0	0	11	16	25	37	19	28	13	19

Reminders and push notifications	App's ability to send push notification reminders when the pregnancy month/trimester begins	2.5	1.1	4	3	27	17	47	30	50	31	31	19	2.7	1.1	2	2	13	14	23	25	28	31	25	27	2.2	1.0	2	3	14	21	24	35	22	32	6	9
	App's ability to schedule reminders for routine activities (e.g., drinking, diapering, feeding, pumping, and sleeping)	2.1	1.1	16	10	26	16	59	37	40	25	18	11	2.2	1.1	7	8	13	14	36	40	25	27	10	11	2.0	1.2	9	13	13	19	23	34	15	22	8	12
	App's ability to change reminders and notifications settings	2.8	1.0	2	1	17	11	36	23	60	38	44	28	2.9	1.0	1	1	9	10	16	18	39	43	26	29	2.7	1.0	1	1	8	12	20	29	21	31	18	26
Notes and records	App's ability to record the latest period date or the expected delivery date	2.9	1.0	2	1	11	7	40	25	52	33	54	34	3.1	1.0	2	2	3	3	18	20	29	32	39	43	2.7	1.0	0	0	8	12	22	32	23	34	15	22
	App's ability to change the expected delivery date following medical re-evaluation	3.0	1.0	2	1	9	6	32	20	57	36	59	37	3.1	0.9	2	2	3	3	13	14	36	40	37	41	2.9	1.0	0	0	6	9	19	28	21	31	22	32
	App's ability to record physiological values of the mother (e.g., pressure, temperature, and mood)	2.9	1.0	4	3	10	6	35	22	54	34	56	35	2.9	1.0	3	3	3	3	23	25	30	33	32	35	2.9	1.0	1	1	7	10	12	18	24	35	24	35
	App's ability to record contractions or kicks	3.0	1.0	2	1	13	8	32	20	49	31	63	40	3.2	1.0	1	1	4	4	17	19	27	30	42	46	2.8	1.1	1	1	9	13	15	22	22	32	21	31
	App's ability to record routine activities of the mother or the newborn (e.g., drinking, steps, diapers changes, bottle feeding, and sleeping patterns/times)	2.3	1.1	10	6	24	15	60	38	45	28	20	13	2.4	1.1	6	7	11	12	32	35	29	32	13	14	2.1	1.0	4	6	13	19	28	41	16	24	7	10
	App's ability to record the medical care the mother or the newborn has received (e.g., medications and vaccination shots)	2.9	1.0	3	2	8	5	43	27	58	36	47	30	2.9	1.0	2	2	5	5	25	27	27	30	32	35	2.8	0.9	1	1	3	4	18	26	31	46	15	22
	App's ability to track the newborn's developmental milestones	2.8	0.9	3	2	8	5	42	26	65	41	41	26	2.9	1.0	2	2	5	5	21	23	36	40	27	30	2.8	0.9	1	1	3	4	21	31	29	43	14	21

	App's ability to record anthropometric measurements of the fetus (e.g., height, weight, and head circumference)	3.0	0.9	2	1	7	4	39	25	55	35	56	35	3.1	1.0	1	1	5	5	18	20	31	34	36	40	2.9	0.9	1	1	2	3	21	31	24	35	20	29
	App's ability to record anthropometric measurements of the newborn (e.g., height, weight, and head circumference)	3.0	1.0	2	1	9	6	34	21	54	34	60	38	3.1	1.0	1	1	6	6	15	16	31	34	38	42	2.9	1.0	1	1	3	4	19	28	23	34	22	32
	App's ability to record measurements of the mother's weight at baseline and during pregnancy	2.6	1.0	4	3	13	8	50	31	62	39	30	19	2.8	1.0	2	2	9	10	19	21	39	43	22	24	2.5	0.9	2	3	4	6	31	46	23	34	8	12
	App's ability to record measurements of the mother's weight in the postnatal period	2.5	1.0	4	3	22	14	48	30	63	40	22	14	2.6	1.0	2	2	13	14	20	22	40	44	16	18	2.3	0.9	2	3	9	13	28	41	23	34	6	9
	App's ability to create a sleep diary for the mother	2.2	1.0	7	4	25	16	64	40	51	32	12	8	2.3	1.0	4	4	12	13	35	38	32	35	8	9	2.1	0.9	3	4	13	19	29	43	19	28	4	6
	App's ability to create a sleep diary for the newborn	2.5	1.0	4	3	21	13	43	27	69	43	22	14	2.6	1.0	3	3	10	11	21	23	42	46	15	16	2.4	0.9	1	1	11	16	22	32	27	40	7	10
Social support	Integration of the app with social networks (e.g., Facebook, Twitter)	1.3	1.2	50	31	42	26	45	28	13	8	9	6	1.6	1.1	16	18	26	29	32	35	12	13	5	5	0.9	1.1	34	50	16	24	13	19	1	1	4	6
	Presence of a FAQ (frequently asked questions) page in the app	2.5	1.0	6	4	22	14	47	30	60	38	24	15	2.5	1.1	6	7	10	11	24	26	36	40	15	16	2.4	0.9	0	0	12	18	23	34	24	35	9	13
	Presence of social mechanisms allowing the user to interact with each other and share experiences (e.g., community, forum, and chat)	2.1	1.2	18	11	33	21	49	31	39	25	20	13	2.3	1.2	8	9	16	18	25	27	26	29	16	18	1.8	1.1	10	15	17	25	24	35	13	19	4	6
	Presence of social mechanisms allowing the user to interact with healthcare staff (e.g., community, forum, and chat)	3.0	1.0	3	2	9	6	25	16	64	40	58	36	3.1	1.0	3	3	4	4	13	14	34	37	37	41	3.0	0.9	0	0	5	7	12	18	30	44	21	31
App techn ical	Authentication request to the user	2.9	1.1	6	4	16	10	25	16	52	33	60	38	2.9	1.2	4	4	9	10	17	19	27	30	34	37	3.0	1.1	2	3	7	10	8	12	25	37	26	38

	Presence of a privacy policy in the app	2.8	1.1	7	4	12	8	35	22	53	33	52	33	2.8	1.1	4	4	4	4	24	26	30	33	29	32	2.8	1.2	3	4	8	12	11	16	23	34	23	34
	If present, availability of a privacy policy properly written in Italian	2.7	1.2	8	5	18	11	33	21	50	31	50	31	2.7	1.2	5	5	8	9	21	23	29	32	28	31	2.7	1.2	3	4	10	15	12	18	21	31	22	32
	Ability for the user to access all app content for free (without any payment)	3.4	0.9	1	1	8	5	8	5	44	28	98	62	3.5	0.8	1	1	3	3	4	4	26	29	57	63	3.4	0.9	0	0	5	7	4	6	18	26	41	60
	Access to full app usage based on specific inclusion criteria (e.g., national health service card, place of living, and certification by a health professional)	2.7	1.1	6	4	19	12	42	26	44	28	48	30	2.7	1.2	5	5	9	10	21	23	28	31	28	31	2.6	1.1	1	1	10	15	21	31	16	24	20	29
	Requirement for the user to sign an informed consent to use the app	2.6	1.2	10	6	23	14	30	19	50	31	46	29	2.7	1.2	7	8	8	9	16	18	30	33	30	33	2.5	1.2	3	4	15	22	14	21	20	29	16	24
	Presence of references about the contents provided through the app	2.6	1.1	5	3	25	16	34	21	52	33	43	27	2.7	1.0	2	2	11	12	21	23	35	38	22	24	2.6	1.2	3	4	14	21	13	19	17	25	21	31
	Presence of a glossary of the most used medical terms	2.9	1.0	4	3	11	7	36	23	52	33	56	35	2.8	1.1	2	2	8	9	25	27	26	29	30	33	3.0	1.0	2	3	3	4	11	16	26	38	26	38
	Declaration (through the provision of specific references) of the scientific responsibility of the contents provided through the app	2.5	1.1	8	5	19	12	47	30	48	30	37	23	2.5	1.1	6	7	8	9	28	31	28	31	21	23	2.5	1.1	2	3	11	16	19	28	20	29	16	24
	Possibility to back-up/restore data within the app	2.5	1.1	7	4	18	11	57	36	43	27	34	21	2.5	1.2	6	7	11	12	28	31	26	29	20	22	2.5	1.0	1	1	7	10	29	43	17	25	14	21
	Possibility to download data collected through the app	2.7	1.0	5	3	12	8	50	31	50	31	42	26	2.7	1.1	4	4	8	9	26	29	27	30	26	29	2.7	0.9	1	1	4	6	24	35	23	34	16	24
	Presence of multilanguage support	2.8	1.1	8	5	12	8	40	25	45	28	54	34	2.8	1.2	5	5	7	8	21	23	26	29	32	35	2.8	1.1	3	4	5	7	19	28	19	28	22	32
	App's ability to geolocate the user to provide more detailed information	2.0	1.2	22	14	34	21	49	31	32	20	22	14	2.0	1.4	16	18	19	21	19	21	21	23	16	18	1.9	1.0	6	9	15	22	30	44	11	16	6	9
	App's ability to book visits, vaccinations, and checkups	3.4	0.8	1	1	5	3	15	9	46	29	92	58	3.4	0.9	1	1	3	3	11	12	24	26	52	57	3.5	0.7	0	0	2	3	4	6	22	32	40	59
	App's ability to update users'	3.0	0.9	2	1	9	6	31	19	66	42	51	32	2.9	1.0	2	2	6	7	17	19	36	40	30	33	3.0	0.8	0	0	3	4	14	21	30	44	21	31

	account preferences																																				
	Use of a simple, informal, and friendly tone by the app	2.9	1.1	4	3	13	8	37	23	50	31	55	35	2.9	1.1	3	3	5	5	23	25	27	30	33	36	2.8	1.1	1	1	8	12	14	21	23	34	22	32
	App's ability to adapt to screen orientation (both portrait and landscape)	2.4	1.2	10	6	27	17	44	28	42	26	36	23	2.4	...1.2	7	8	16	18	24	26	22	24	22	24	2.5	1.1	3	4	11	16	20	29	20	29	14	21
	App's ability to learn user's preferences over time	2.3	1.2	11	7	30	19	48	30	41	26	29	18	2.4	1.2	8	9	13	14	26	29	24	26	20	22	2.2	1.1	3	4	17	25	22	32	17	25	9	13
	App's ability to implement intuitive and predictable navigation patterns	3.2	0.9	0	0	7	4	28	18	55	35	69	43	3.2	0.9	0	0	4	4	13	14	32	35	42	46	3.1	0.9	0	0	3	4	15	22	23	34	27	40
	Presence of app contents validated by an institutional source (local, regional, or national)	2.9	1.0	2	1	13	8	38	24	52	33	54	34	2.9	1.1	2	2	9	10	17	19	31	34	32	35	2.9	0.9	0	0	4	6	21	31	21	31	22	32
	Presence of certification of the app as a medical device according to Italian law	2.7	1.2	5	3	22	14	39	25	39	25	54	34	2.7	1.2	4	4	13	14	18	20	24	26	32	35	2.7	1.1	1	1	9	13	21	31	15	22	22	32
	App's ability to provide contents through different ways (e.g., text, video, audio)	2.7	1.0	4	3	12	8	53	33	46	29	44	28	2.7	1.1	4	4	7	8	25	27	27	30	28	31	2.7	0.9	0	0	5	7	28	41	19	28	16	24
	App's ability to ask about user satisfaction	2.4	1.1	8	5	22	14	57	36	38	24	34	21	2.5	1.2	5	5	12	13	30	33	20	22	24	26	2.3	1.0	3	4	10	15	27	40	18	26	10	15
